# Humoral and Cellular Response Following Vaccination With the BNT162b2 mRNA COVID-19 Vaccine in Patients Affected by Primary Immunodeficiencies

**DOI:** 10.3389/fimmu.2021.727850

**Published:** 2021-10-04

**Authors:** Donato Amodio, Alessandra Ruggiero, Mayla Sgrulletti, Chiara Pighi, Nicola Cotugno, Chiara Medri, Elena Morrocchi, Luna Colagrossi, Cristina Russo, Salvatore Zaffina, Gigliola Di Matteo, Cristina Cifaldi, Silvia Di Cesare, Beatrice Rivalta, Lucia Pacillo, Veronica Santilli, Carmela Giancotta, Emma Concetta Manno, Marta Ciofi Degli Atti, Massimiliano Raponi, Paolo Rossi, Andrea Finocchi, Caterina Cancrini, Carlo Federico Perno, Viviana Moschese, Paolo Palma

**Affiliations:** ^1^ Academic Department of Pediatrics (DPUO), Research Unit of Clinical Immunology and Vaccinology, Bambino Gesù Children’s Hospital, Istituto di Ricovero e Cura a Carattere Scientifico (IRCCS), Rome, Italy; ^2^ Department of Neuroscience, Biomedicine and Movement Sciences, University of Verona, Verona, Italy; ^3^ Chair of Pediatrics, Department of Systems Medicine, University of Rome “Tor Vergata”, Rome, Italy; ^4^ Pediatric Immunopathology and Allergology Unit, Policlinico Tor Vergata, Rome, Italy; ^5^ PhD Program in Immunology, Molecular Medicine and Applied Biotechnology, University of Rome Tor Vergata, Rome, Italy; ^6^ Microbiology and Diagnostic Immunology Unit, Bambino Gesù Children’s Hospital, Istituto di Ricovero e Cura a Carattere Scientifico (IRCCS), Rome, Italy; ^7^ Occupational Medicine Unit, Bambino Gesù Children’s Hospital, Istituto di Ricovero e Cura a Carattere Scientifico (IRCCS), Rome, Italy; ^8^ Academic Department of Pediatrics (DPUO), Immune and Infectious Diseases Division, Research Unit of Primary Immunodeficiencies, Bambino Gesù Children’s Hospital, Istituto di Ricovero e Cura a Carattere Scientifico (IRCCS), Rome, Italy; ^9^ Clinical Pathways and Epidemiology Unit—Medical Direction, Bambino Gesù Children’s Hospital, Istituto di Ricovero e Cura a Carattere Scientifico (IRCCS), Rome, Italy; ^10^ Medical Direction, Bambino Gesù Children’s Hospital, Istituto di Ricovero e Cura a Carattere Scientifico (IRCCS), Rome, Italy; ^11^ Multimodal Medicine Research Area, Bambino Gesù Children’s Hospital, Istituto di Ricovero e Cura a Carattere Scientifico (IRCCS), Rome, Italy; ^12^ UniCamillus—Saint Camillus International University of Health Sciences, Rome, Italy

**Keywords:** BNT162b2 mRNA COVID-19 vaccine, Comirnaty, SARS-CoV-2, COVID-19, inborn errors of immunity, vaccine efficacy, antigen-specific T cell, SARS-CoV-2 antibody

## Abstract

Mass SARS-Cov-2 vaccination campaign represents the only strategy to defeat the global pandemic we are facing. Immunocompromised patients represent a vulnerable population at high risk of developing severe COVID-19 and thus should be prioritized in the vaccination programs and in the study of the vaccine efficacy. Nevertheless, most data on efficacy and safety of the available vaccines derive from trials conducted on healthy individuals; hence, studies on immunogenicity of SARS-CoV2 vaccines in such populations are deeply needed. Here, we perform an observational longitudinal study analyzing the humoral and cellular response following the BNT162b2 mRNA COVID-19 vaccine in a cohort of patients affected by inborn errors of immunity (IEI) compared to healthy controls (HC). We show that both IEI and HC groups experienced a significant increase in anti-SARS-CoV-2 Abs 1 week after the second scheduled dose as well as an overall statistically significant expansion of the Ag-specific CD4+CD40L+ T cells in both HC and IEI. Five IEI patients did not develop any specific CD4+CD40L+ T cellular response, with one of these patients unable to also mount any humoral response. These data raise immunologic concerns about using Ab response as a sole metric of protective immunity following vaccination for SARS-CoV-2. Taken together, these findings suggest that evaluation of vaccine-induced immunity in this subpopulation should also include quantification of Ag-specific T cells.

## Introduction

Since the rapid spread of COVID-19 across the globe and the identification of SARS-CoV-2 genomic sequence, enormous international scientific and economic efforts have been made to develop safe and effective vaccines. In fact, in the absence of a specific treatment against the SARS-CoV-2, except for monoclonal antibodies that are licensed for few selected clinical conditions, the only strategy to combat the virus and control the pandemic is to vaccinate the population (1, 2). To date, in Europe, four vaccines against SARS-CoV-2 have been approved by the EMA agency; two mRNA vaccines and two attenuated adenovirus vector vaccines. Despite this, the vaccination campaign is proceeding in a non-homogeneous manner with significant differences among countries. The identification of at-risk categories led to prior vaccine administration to vulnerable populations, especially elderly and patients with comorbidities.

Individuals with inborn errors of immunity (IEI) have an increased susceptibility to infections that often affects the clinical outcome; thus, routine immunization represents a critical issue in this population and a precise vaccine schedule is recommended (3). Indeed, vaccine response may vary depending on the type of immune disorder; however, with caution for live attenuated vaccines where data are limited, an overall protective effect has been demonstrated with significant reduction of morbidity and mortality and of healthcare cost containment.

Theoretically, primary immunodeficient patients are assumed to be at high risk of developing severe COVID-19. Most studies have described the course of SARS-CoV-2 infection in antibody deficiency (4–8). More recently, Meyts et al. described a large international cohort of children and adults with IEI mostly experiencing a mild course of disease, although a higher frequency of young individuals admitted to ICU compared to the general population was observed (9).

Since IEI consist of more than 450 monogenic defects, and these reports only partially cover the broad spectrum of IEI disorders (10, 11).

In accordance with interim indications for primary immunodeficient patients (3), COVID-19 vaccines should be advised according to national vaccine schedule, unless contraindicated. IEI patients are characterized by a generally reduced or completely absent vaccine response, depending on the type of immune disorders (12). Given the heterogeneity of IEI disorders with various degrees of immune impairment, it is not possible to define general recommendations regarding immunization. As for routine immunization, it is reasonable to speculate that in these patients, the anti-SARS-CoV-2 vaccine response might be suboptimal, due to the impaired immune system. Thus, vaccination schedule in such vulnerable population needs an accurate assessment of risk-benefits to grant both the best possible protection and avoid unnecessary adverse events. Additional knowledge on the safety and effectiveness of SARS-CoV-2 vaccines in such vulnerable population is paramount since most data come from healthy subjects (13, 14). Indeed, in-depth immunological evaluations following SARS-CoV-2 vaccination might establish correlates of protection other than SARS-CoV-2-specific serology and translate these data for the benefits of other cohorts, i.e., transplanted patients and/or patients receiving chemotherapy, immunosuppressive therapies, or biologic response modifiers.

Given the lack of information on the safety and effectiveness of vaccines in general and anti-SARS-CoV-2 in particular in IEI patients, in this work, we seek to describe the effect of the BNT162b2 mRNA COVID-19 vaccine in this cohort. We first analyzed the humoral response by the mean of two different assays. Despite the development of the humoral response following vaccination, we deeply explored the cellular response by focusing on the SARS-CoV-2-specific CD4+ T cells, which are known to be fundamental for the production of effective neutralizing antibodies (Abs) in both convalescent adults and children and vaccinated healthy controls (HC) (15–17).

## Materials and Methods

### Study Participants

Twenty-one patients with IEI were enrolled from February to March 2021 at Bambino Gesù Children’s Hospital and Tor Vergata University Hospital, in Rome (**Table 1**). According to national regulations, only Latium region residents were eligible for vaccination in our centers with few exceptions. This prospective observational study included patients aged 16–59 years affected by IEI, according to ESID criteria (19). All patients were naïve to SARS-CoV-2 infections as demonstrated by the absence of SARS-CoV-2 Abs both anti-spike and anti-nucleocapsid protein and received the BNT162b2 mRNA COVID-19 vaccine, with a schedule of two doses of 30 μg 21 days apart (20). Longitudinal blood samples were collected on day of vaccination (D0), 21 days after the first dose (D21), and 7 days after the second dose (D28). All patients had negative serology and/or molecular tests for SARS-CoV-2 by nasopharyngeal swabs prior to vaccination. HC with no comorbidities aged <60 who received the BNT162b2 mRNA COVID-19 vaccine were also investigated. Healthy vaccinated gender-matched donors were used as controls. HC were older than IEI (*p* = 0.003 and *p* = 0.0001 for cellular and humoral analysis, respectively, **Table 2**). All participants received a survey reporting any adverse events and side effects following each dose of vaccine.

**Table 1:** Demographics, diagnosis, and clinical and genetic data of IEI cohort.

Pt code	Gender (M/F)	Age (years)	Diagnosis	Clinical phenotype	Comorbidities	Genetics	IVIG(Y/N)	Other treatments	Past treatments	Vaccine side effects
1	M	45	XLA	LRTI; skin infections;	COPD, chronic pancreatitis, sclerosing cholangitis	Hemizygous *BTK* missense VoUS: c.1078 A>G, p.T316A	Y	Antibiotic prophylaxis; ICS-LABA	/	Myalgia
2	F	48	CVID	URTI; LRTI; UTI; vaginal candidiasis; Rheumatoid Arthritis;	Headache; Fibromyalgia;	Heterozygous *IKBKB* missense VoUS: c.1465A>G: p.S489G	Y	Sarilumab, SSZ	Anti-TNF agents, RTX - DMARDS	Fever
3	M	32	unPAD	URTI; LRTI; Rheumatoid Arthritis	/	Negative *	Y	SSZ, Hydroxychloroquine	Anti-TNF agents, RTX - DMARDS	Fever and myalgia
4	M	51	unPAD	URTI; LRTI; past gastric non-Hodgkin lymphoma	Asthma	Negative *	Y	/	R-CHOP chemotherapy (2006)	Fever and malaise
5	M	18	CVID	Past thrombocytopenia and neutropenia; URTI; LRTI	/	Negative **	Y	/	Parenteral corticosteroids and high dose IVIG	Fever and malaise
6	F	32	WHIM-like	HPV infections	Pelvic inflammatory disease	WES in progress	N	/	/	Local pain
7	F	51	CVID	LRTI; chronic sinusitis; *S. epidermidis* superinfection on surgical wound; ulcerative colitis and spondyloarthritis	Asthma	Heterozygous *NFKB1* missense VoUS: c.1501C>G:p.L501V heterozygous *TCF3* missense VoUS:c.931G>C: p.V311L and c.920A>G: p.H307R	Y	SSZ	Betamethasone, methylprednisolone	Myalgia
8	M	34	CVID	GI; UTI; arthritis	/	Negative ***	Y	/	/	Not referred
9	M	20	unPAD	URTI	Allergic rhinitis, headache, anxiety disorder	Negative ***	N	Antihistamine; valproic acid	/	Malaise and fever
10	F	21	CVID	ITP; Hashimoto thyroiditis	Allergic rhinitis	Negative *	Y	Antihistamine	Parenteral corticosteroids, high-dose IVIG, MMF	Not reported
11	F	31	CVID	Past ITP	/	Heterozygous *PTPN22* missense VoUS: c.1858C>T p.R620W	Y	/	Parenteral CCS, high dose IVIG, RTX	Fever
12	F	38	CVID	Vitiligo; Hashimoto thyroiditis	/	Heterozygous *TNFRSF13B* (TACI) missense VoUS: c.512T>G; p.L171R	Y	/	/	Not referred
13	F	25	CVID	GI; URTI; HP infection; recurrent abdominal pain	Chronic sinusitis	Heterozygous *TNFRSF13B* (TACI) missense VoUS: c.58C>T; p.R20C	Y	/	/	Fever, myalgia and headache
14	F	59	unPAD	Legionella pneumonia;	Chronic renal failure, hypertension, obesity	CVID NGS panel in progress	Y	Antibiotic prophylaxis	/	Not referred
15	F	33	unPAD	URTI; LRTI;	Allergic rhinitis and asthma	NGS analysis progress	N	ICS-LABA; antihistamine	NO	Fever
16	M	16	CVID	Past Burkitt lymphoma; URTI, LRTI	/	NGS analysis progress	Y	/	R-CODOX M chemotherapy	Local pain
17	M	21	CVID	LRTI; chronic sinusitis; mild asymptomatic thrombocytopenia	Severe hypermetropia; psychomotor delay, dysgenesis of corpus callosum, cysts of arachnoid mater	WGS in progress	Y	/	/	Not referred
18	M	21	CVID	URTI; recurrent laryngospasm; splenomegaly; atypical mycobacterial infection; ITP	Preclinical diabetes; symmetric axonal sensitive polyneuropathy	Heterozygous *TNFRSF13C* (BAFFR) missense VoUS: c.C475T:p.H159Y	Y	/	/	Not referred
19	M	20	CVID	URTI; chronic rhinosinusitis; Hashimoto thyroiditis	Obsessive–compulsive disorder	Novel unpublished *NFKB1* variant: functional test ongoing	N	/	/	Not referred
20	M	21	CVID	ITP; LRTI; URTI; bronchiectasis; bone marrow hypoplasia; mycobacterial infection; vitiligo	/	Novel unpublished *NFKB1* variant: functional test ongoing	Y	/	RTX	Not referred
21	F	36	CVID	Thrombocytopenia	Past melanoma	Heterozygous *CTLA4* missense VoUS: c.224G>A; p.R75Q and heterozygous *PTEN* missense VoUS: c.596T>C p.M199T	N	/	/	Rhinitis and sore throat

XLA, X-linked agammaglobulinemia; unPAD, unclassified antibody deficiency; LRTI, lower respiratory tract infection; COPD, chronic obstructive pulmonary disease; ICS-LABA, inhaled corticosteroid and long-acting β2-agonist; CVID, common variable immunodeficiency; URTI, upper respiratory tract infection; UTI urinary tract infection; SSZ, sulfasalazine; Anti-TNF agents, anti-tumor necrosis factor agents; DMARDs, disease-modifying anti-rheumatic drugs; RTX, rituximab; R-CHOP chemotherapy, (Rituximab, cyclophosphamide, doxorubicin, vincristine, and prednisone) chemotherapy; IVIG, intravenous immunoglobulin; HPV, human papilloma virus; ITP, immune thrombocytopenia; MMF, mycophenolate mofetil; GI, gastrointestinal infection; HP, Helicobacter pylori; R-CODOX M, R-Rituximab C—cyclophosphamide and cytarabine O—vincristine, also known as oncovin DOX—doxorubicin M—methotrexate chemotherapy.

*CVID NGS panel available on Cifaldi et al., 2019 https://doi.org/10.3389/fimmu.2019.00316.

**CID NGS panel available on Cifaldi et al., 2019 https://doi.org/10.3389/fimmu.2019.00316.

***Haloplex NGS panel available on Cifaldi et al., 2019 https://doi.org/10.3389/fimmu.2019.00316 (18).

**Table 2:** Cohort characteristics.

	HC—cellular response (*N* = 65)	HC—humoral response (*N* = 18)	IEI	*p*-value
Age, years (mean)	43.7	45.0	32.0	*p* = 0.003 HC cell *vs.* IEI, *p* < 0.0001 HC humoral *vs.* IEI
Sex (M/F)	7/11	19/46	11/10	ns
Ethnicity	All Caucasian	All Caucasian	All Caucasian	ns

ns, not significant.

All procedures performed in the study were in accordance with the ethical standards of the institutional research committee and with the 1964 Helsinki declaration and its later amendments or comparable ethical standards. A local ethical committee approved the study and written informed consent was obtained from all participants or legal guardians. Age, gender, clinical, and routine laboratory characteristics of the cohort are described in **Table 1**.

### Sample Collection and Storage

Venous blood was collected in EDTA tubes and processed within 2 h. Plasma was isolated from blood and stored at −80°C. Peripheral blood mononuclear cells (PBMCs) were isolated from blood of all patients with Ficoll density gradient and cryopreserved in FBS 10% DMSO until analysis, in liquid nitrogen.

### Humoral Response

For serology test, we used different chemiluminescence test performed on an automated analyzer following the manufacturer’s instructions.

Anti-SARS CoV-2 IgG Ab titers were measured at D0, D21, and D28. In particular, we measured Abs against the S1-receptor-binding-domain (RBD) (Roche, cutoff: 0.8 U/ml) and anti-trimeric SARS-CoV-2 Ab (LIAISON^®^ SARS-C0V-2 DiaSorin, cutoff: 13 AU/ml).

The LIAISON^®^ SARS-CoV-2 TrimericS IgG (DiaSorin—Saluggia TO) is an indirect chemiluminescent immunoassay (CLIA) intended for the qualitative and semi-quantitative detection of anti-trimeric spike protein specific IgG antibodies to SARS-CoV-2 in human serum, used on the LIAISON^®^ XL platform Analyzer. The test detects IgG antibodies against the Trimeric complex, which includes the RBD and NTD sites from the three subunit S1 (the Trimeric complex). Test results are reported as positive or negative along with a numeric value for semi-quantitative measurement for values between 13 AU/ml and 800 AU/ml. TrimericS IgG assay has a quantification range between 4.81 BAU/ml and 2,080 BAU/ml (dilution factor 1:20).

Elecsys anti- SARS-CoV-2 and Elecsys anti-SARS-CoV-2 S (Roche Diagnostics) test on a Cobas e801 analyzer have been used.

The Elecsys^®^ Anti-SARS-CoV-2 is an immunoassay for the *in vitro* qualitative detection of a mix of antibodies (including IgA, IgM, and IgG) to SARS-CoV-2 in human serum and plasma. In order to investigate a broad-spectrum immune response, we use two types of Roche antibody assays using a recombinant protein, respectively, for the S antigen and for the nucleocapsid (N) antigen in a double-antigen sandwich assay format. Results for anti-N antibodies are expressed as “present” or “absent” on the basis of a cutoff index (COI) ≥ 1.0 and COI < 1.0, respectively. Titer for Anti-S Ab was interpreted as absent when <0.8 U/ml (<0.8 BAU/ml) and as present when ≥0.80 U/ml (≥0.8 BAU/ml). When antibody titer was higher than 250 U/ml (250 BAU/ml), the instrument automatically executed a 20-fold dilution, ranging the upper limit of quantification to 5,000 U/ml (5,000 BAU/ml).

### CD4 Ag-Specific T-Cell and B-Cell Phenotype

SARS-CoV-2-specific CD4+CD40L+ T cells were identified, as previously described (17). Briefly, thawed PBMCs were plated (1.5 × 10^6^/aliquot/200 μl) in 96-well plates containing CD154-PE (CD40L, BD PharMingen, Franklin Lakes, NJ, USA) and anti-CD28 (1 mg/ml) in the presence or absence of 0.4 mg/ml PepTivator SARS-CoV-2 Prot_S (Miltenyi Biotec, Bergisch Gladbach, Germany). Following 16 h incubation at 37°C/5% CO_2_, PBMCs were centrifuged and stained with LIVE/DEAD fixable NEAR-IR dead cell stain kit (for 633 or 635 nm excitation, ThermoFisher, Waltham, MA, USA) 1 μl per 10^6^cells/ml for 15 min at room temperature (RT), protected by light. Surface staining was performed using the following antibodies: CD3 PE-CF594 [clone UCHT1, BD (562280)], CD4 APC-Cy7 [clone RPA-T4, BD (557871)], CD27 FITC [clone M-T271, BD (555440)], CD45RO PE-Cy5 (clone UCHL1, BD), CD185 BV605 (CXCR5, clone RF8B2, BS), CD10 BV510 (clone HI10a, BD 563032), CD19 APC-R700 (clone SJ25C1, BD 659121), CD21 APC (clone B-Ly4, BD 559867), and IgD BV421 (clone IA6-2, BD 565940). T- and B-cell population and SARS-CoV-2-specific CD40L+CD4+ T cells were gated as previously reported (17).

Due to limited sample available for testing and in accordance with the evidence of a Th1 response following both the disease and vaccination and our previous work showing that interaction between CD4 T cell and B cell is critical in order to mount specific neutralizing antibodies, we decided to focus our efforts on CD4 T-cell response (17, 20–22).

### Quantification and Statistical Analysis

Statistical analyses were performed using GraphPad Prism 8 (GraphPad Software, Inc., San Diego, CA). Statistical significance was set at *p* < 0.05, and the test was two-tailed. All data were analysed by D’Agostino-Pearson to assess normality. As indicated in figure legends, paired and non-paired non-parametric tests were used to assess differences between Ab load at the different time points, and between HC and IEI, respectively. Spearman’s correlation was used to compute the association between variables. GraphPad Prism 8 software was used for statistical analysis of cell-type distribution and serological parameters for demographic and routine laboratory blood tests.

## Results

### Study Population

The study included 21 IEI patients aged 16–59 years (mean age, 32 years). Patient samples were collected right before the first vaccine dose (D0), for baseline immunological investigation, at the second dose (D21), and 1 week after the second dose (D28). In line with previous evidence showing an increase in specific ab titer and cellular responses 7 days following the second dose (20, 23), we decided to consider the same time points. Demographics, diagnosis, and clinical and genetic data are reported in **Table 1**. The study cohort included patients affected by common variable immunodeficiency (CVID, *n* = 14) and unclassified antibody deficiency (unPAD, *n* = 5) and two patients affected by X-linked agammaglobulinemia (XLA) and WHIM-like disorder, respectively (**Table 1**). Recurrent infections represented the most common clinical manifestations of these patients (17/21, 81%), followed by autoimmunity, mainly cytopenias, arthritis, and autoimmune thyroiditis, (13/21, 62%) and neoplasia (3/21, 14%). Allergic diseases, such as allergic rhinitis and asthma, represented common comorbidities. Sixteen out of 21 patients (76%) are currently receiving Ig replacement therapy and 6/21 (28%) have been treated or are still being treated with immunosuppressant drugs or biologics. Two patients previously received chemotherapy due to neoplasia. Routine immunological evaluation at baseline is reported in **Table 3**. Healthy vaccinated age- and gender-matched donors were used as controls.

**Table 3:** Routine immunological evaluation at baseline.

	Pt1	Pt2	Pt3	Pt4	Pt5	Pt6	Pt7	Pt8	Pt9	Pt10	Pt11
**Hb (mg/dl)**	15.1	16.9	15.5	14.3	15.2	12.9	14.1	15.4	15.2	12.2	14.4
**PLT (10x3/ml)**	123	174	214	245	129	228	259	165	218	253	192
**WBC (10x3/ml)**	9.07	6.22	7.6	5.39	4.06	9.01	4.76	4.42	5.79	3.73	4.52
**Eosinophils (% and 10x3/ml)**	1.3/120	2.9/180	2.5/190	0.4/20	4.4/180	7.3/660	1.3/60	2.3/100	5.7/330	1.1/40	3.5/220
**Total lymphocytes (10x3/ml)**	1.05	2.76	2.6	2.7	1.6	4.8	2.2	1.2	2.3	1.2	1.1
**CD3+ (% and cell/µl)**	87/913	81/2235	83/2158	64.7/1746	77/1232	86/4128	58/1276	70/840	70/1610	73/876	82.7/909
**CD4+ (% and cell/ µl)**	46/483	65/1794	36/936	37/999	27/432	38/1824	36.7/807	48/576	37/851	40.6/487	39.7/436
**CD8+ (% and cell/ µl)**	41/430	15/414	44/1144	24/648	45/720	31.7/1521	17/374	20/240	25/575	17/204	42.1/463
**CD19+ (% and cell/µl)**	5.5/52	9/248	6.7/174	17/459	12/192	6.3/302	26/572	18/216	17/391	19.8/237	8/88
**CD16+56+ (% and cell/µl)**	6.5/68	8.8/243	9.9/257	15.6/421	7/112	7.8/374	8.7/191	11/132	9/207	12.2/146	6.1/66
**CD3+CD4+CD27+CD45RO- Naïve T cell (%)**	26	68.6	52.9	64.2	41.9	79	70.5	66.5	70	27.2	48
**CD3+CD4+CD27+CD45RO+ Tcm (%)**	49.9	25.4	33.2	25.9	49.1	12.2	22.2	26.8	21.7	68.6	41.1
**CD3+CD4+CD27-CD45RO+ Tem (%)**	4.52	1.43	1.14	1.98	2.54	3.79	1.13	1.94	2.73	0.31	1.88
**CD3+CD4+CD27-CD45RO- Temra (%)**	17.2	2.37	8.74	6.08	5.5	3.93	3.11	2.99	3.54	2.73	6.59
**CD3+CD4+CD27+CD45RO+CXCR5+ pTfh (%)**	28.9	24.9	18.7	24.3	45.5	8.22	23.3	12.8	17	53.9	30.3
**CD27-IgD- Double negative B cells (%)**	16.5	0.86	2.11	6.61	1.13	3.77	2.58	8.29	8.62	7.48	1.56
**CD27+IgD+ Unswitched memory B cells (%)**	3.46	0.39	0.56	11.9	3.46	4.99	0.85	9.73	11.8	14	2.23
**CD27+IgD- Switched memory B cells (%)**	13.8	0.054	0.25	8.11	0.13	5.5	1.26	5.19	5.49	4.16	0.83
**CD27-CD21+ Naïve B cells (%)**	50.4	96.7	85.1	66.4	83.6	34.6	91.5	63	62.6	37.6	90.5
**CD27-CD21- TLM B cells (%)**	27.1	1.75	13.5	5.59	10.2	49	5.22	13	10.8	33.9	4.27
**CD21+ (RM) Switched memory B cells (%)**	76.2	67.9	70.3	84.6	72.5	94.2	84.7	88.1	90.1	62.9	70.3
**CD21- (AM) Switched memory B cells (%)**	19.5	32.1	26.6	10.4	26.4	4.79	13.2	8.62	7.86	33	27.7
**IgG (mg/dl)**	1090	993	723	1420	1132	1132	1165	1159	698	1180	708
**IgA (mg/dl)**	33	50	22	5	5	356	113	45	91	290	45
**IgM (mg/dl)**	10	7	55	131	5	284	45	23	24	25	23
**IgE (kU/L)**	1	1.67	9.94	142	1	2.5	2	3	207	2.47	1
**IgG anti-tet**	NA	NA	NA	R	NR	R	R	TR	R	NA	TR
**IgG anti-pneumo**	NA	NA	NA	R	NR	R	R	TR	R	NA	TR
**TCR α/β**	NA	96.5%	98.3%	96.5%	89.6%	78.2%	91.6%	96.4%	80.8%	89.6%	96%
**TCR γ/δ**	NA	3.3%	1.5%	3%	8.6%	21.5%	6.4%	1.2%	17.7%	10.2%	3.6%
**continued**	**Pt12**	**Pt13**	**Pt14**	**Pt15**	**Pt16**	**Pt17**	**Pt18**	**Pt19**	**Pt20**	**Pt21**	
**Hb (mg/dl)**	13.4	13.3	13.8	12.1	15.6	13.8	14.2	16.3	13	12.9	
**PLT (10x3/ml)**	241	169	339	218	232	109	129	173	84	128	
**WBC (10x3/ml)**	3.81	4.82	7.95	7.6	5.15	7.00	5.73	4.99	2	4.61	
**Eosinophils (% and 10x3/ml)**	0.5/20	1.7/80	1.5/120	2.2/170	2/100	1.2/80	1.9/108	2/100	0.7/14	4/0.18	
**Total lymphocytes (10x3/ml)**	0.8	1.6	2.1	2.0	1.8	2290	1.19	1.65	0.51	0.83	
**CD3+ (% and cell/µl)**	75/600	61.3/981	90.6/1902	88.5/1770	85/1530	90.6/2074	87/1035	83.7/1981	96.6/493	70.9/586	
**CD4+ (% and cell/ µl)**	51/408	35.2/563	53.7/1127	57/1140	30/540	54/1236	50.3/599	41.8/690	56.2/287	43.5/361	
**CD8+ (% and cell/ µl)**	17/136	20/320	29.7/623	25.5/510	43/774	32.5/744	32/381	24.4/403	35.2/179	25/207	
**CD19+ (% and cell/µl)**	10.5/84	11.3/176	3.9/82	9.8/196	6.3/113	1.4/32	3.1/178	9.4/155	0.2/1	13.4/111	
**CD16+56+ (% and cell/µl)**	11.6/93	26.2/419	5.1/107	3.7/74	7.5%/135	7.4/169	9.8/561	6.2/102	2.7/14	14.4/119	
**CD3+CD4+CD27+CD45RO- Naïve T cell (%)**	55.9	46.2	24.5	36.9	51.1	68.8	26.5	81	49.4	32.2	
**CD3+CD4+CD27+CD45RO+ Tcm (%)**	38.6	47.5	46	44.3	23.8	24.4	56.7	16.2	37.6	57.3	
**CD3+CD4+CD27-CD45RO+ Tem (%)**	0.088	0.12	0.5	2.64	0.61	0.47	0.97	1.46	1.21	0.37	
**CD3+CD4+CD27-CD45RO- Temra (%)**	3.22	2.72	26.6	12.9	23.4	4.74	13.4	0.55	10.2	8.46	
**CD3+CD4+CD27+CD45RO+CXCR5+ pTfh (%)**	30.2	38.1	19.2	23.4	18.5	15	30.2	15.1	14.7	28	
**CD27-IgD- Double negative B cells (%)**	5.42	5.79	9.72	15.5	3.87	3.85	1.2	3.27	33.3	4.77	
**CD27+IgD+ Unswitched memory B cells (%)**	4.18	4.9	6.63	3.31	3.57	0.41	3.16	2	0	6.57	
**CD27+IgD- Switched memory B cells (%)**	2.26	1.52	12.7	10	0.84	0.36	0.094	0.8	0	0.72	
**CD27-CD21+ Naïve B cells (%)**	82.1	74.7	57.2	62.9	85.2	91.6	69.4	87.9	33.3	31.9	
**CD27-CD21- TLM B cells (%)**	6.32	13.4	15.9	14.4	7.39	5.22	23.1	5.89	33.3	53.7	
**CD21+ (RM) Switched memory B cells (%)**	72	77.9	88.8	74	81.3	73.3	50	66.9	0	79.1	
**CD21- (AM) Switched memory B cells (%)**	21	16.3	8.71	19.4	15.7	13.3	50	21	0	19.4	
**IgG (mg/dl)**	1021	1197	655	618	1052	876	325	726	746	289	
**IgA (mg/dl)**	5	16	550	96	5	<4	<4	91	<4	17	
**IgM (mg/dl)**	8	34	87	189	25	<5	10	87	<5	37	
**IgE (kU/L)**	1	1	3.78	211	92	NA	NA	NA	2	NA	
**IgG anti-tet**	R	R	NA	NA	NR	NA	NA	R	NA	TR	
**IgG anti-pneumo**	NR	NR	NA	R	NR	NA	NA	R	NA	R	
**TCR α/β**	97.5%	93.4%	92.5%	97%	87.6%	NA	96.9%	83.6%	94.6%	92.3%	
**TCR γ/δ**	3.3%	5.3%	5.9%	2.8%	12.1%	NA	2.3%	9.8%	3.4%	2.3%	

WBC, White blood cells; PLT, Platelets; HB, Hemoglobin; Tcm, central memory T cells; Tem, effector memory T cells; Temra, terminally differentiated effector memory T cells; pTfh, peripheral follicular helper memory T cells; TLM, tissue like memory B-cells; RM, resting memory B-cells; AM, activated memory B-cells.

NA, Not available; NR, Not Responder; R, Responder; TR, Transient Responder.

Serum immunoglobulin concentrations from Whelan MA et al., J. Clin Immunol 2006 (24); T-cell subsets from Schatorie E.J.H. et al., Clin Immunol 2011 (25); B-cell subsets from Piatosa B. et al., Cytometry part B, Clinical Cytometry 2010 and Duchamp M et al., Immunity, Inflammation and Disease 2014 (26, 27); Regulatory T-cell subsets from van Gent R. et al., Clinical Immunology 2009 (28).

### Vaccine Side Effects

As reported in **Table 1**, no severe adverse events following vaccination have been observed. The most common side effect was fever (8/21, 38%), followed by myalgia and malaise (4/21, 19% and 3/21, 14% respectively). Local pain at the site of injection was reported by two patients (9%) and headache by one patient (5%).

### Humoral Response

**Figure 1:**
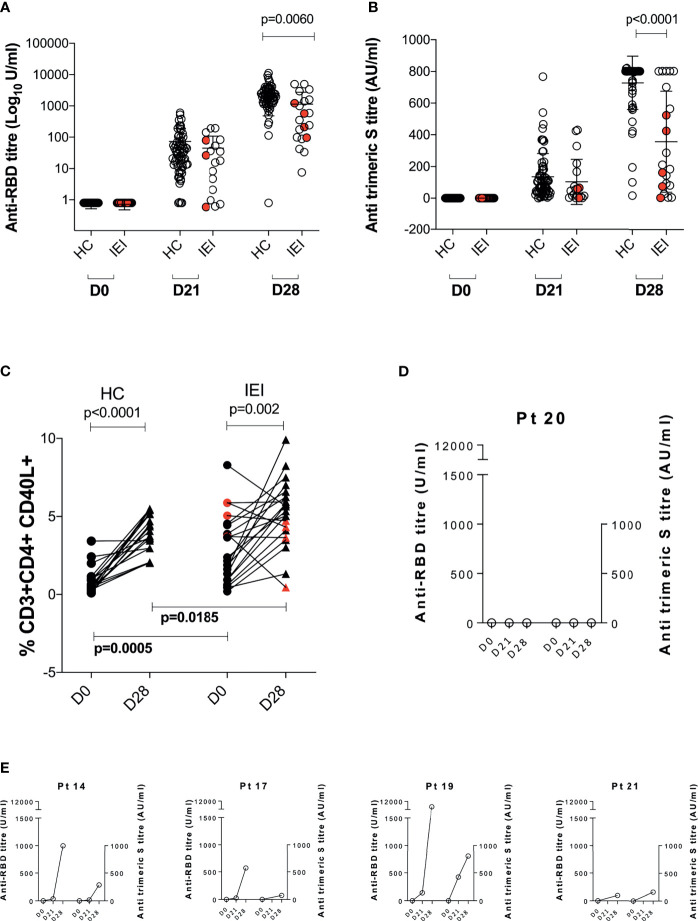
Anti-SARS-CoV-2 immune response before (D0) and at 21 days (D21) after the 1° dose and at 7 days (D28) after the 2° dose of the BNT162b2 mRNA COVID-19 vaccine. Humoral response is represented in **(A)** anti-RBD Ab (Log_10_ U/ml) and **(B)** anti-trimeric S titer (AU/ml). Cellular response is depicted in **(C)**. Humoral response of five patients lacking any cellular response is reported in **(D, E)**. Non-paired non-parametric Mann–Whitney test was used in **(A, B)**; paired non-parametric Wilcoxon tests were used to assess differences between D0 and D28 in HC and IEI in **(C)**. HC, healthy controls; IEI, inborn errors of immunity patients.

We evaluated the humoral response before (D0) and after the first (D21) and second dose (D28, 1 week after the second dose administration) of vaccine in both IEI and HC. Results are summarized in **Figures 1A, B**. Overall, both HC and IEI groups experienced an increase in anti-SARS-CoV-2 Abs between D21 and D28, with only one patient for each category lacking anti-RBD Abs at D28. At D21, Ab levels were similar in the two groups, while at D28, patients with IEI showed lower median specific antibody levels measured as both anti-RBD (**Figure 1A**) and anti-trimeric Abs (**Figure 1B**). In particular, HC showed a higher increase in both anti-RBD titer and anti-trimeric S titer compared to IEI, *p* = 0.0060 and *p* < 0.0001 respectively. Of note, at the end of vaccine schedule at D28, 3/21 (14%) IEI patients had undetectable levels of anti-trimeric Abs, whereas all HC had measurable levels (**Figure 1B**).

### Cellular Response

#### T-Cell Response

Gating strategy for SARS-COV-2-specific T cells (CD4+CD40L+) is summarized in **Supplementary Figure S1**. When we evaluated the Ag-specific cellular response at D0 and D28, we found an overall statistically significant expansion of the CD4+CD40L+ T cells in both HC (*p* < 0.001) and IEI (*p* = 0.002) patients (**Figure 1C**), with different levels at baseline (D0) and D28 between the two groups. However, in 5/21 (24%, Pt14, Pt17, Pt19, Pt20, and Pt21) IEI patients, no increase in the proportion of the Ag-specific T cells could be observed. In one of these five non-responsive patients, Pt20, humoral correlates were also lacking (**Figure 1D**). The remaining four out of five seroconverted at similar levels to HC (**Figure 1E**).

**Figure 2:**
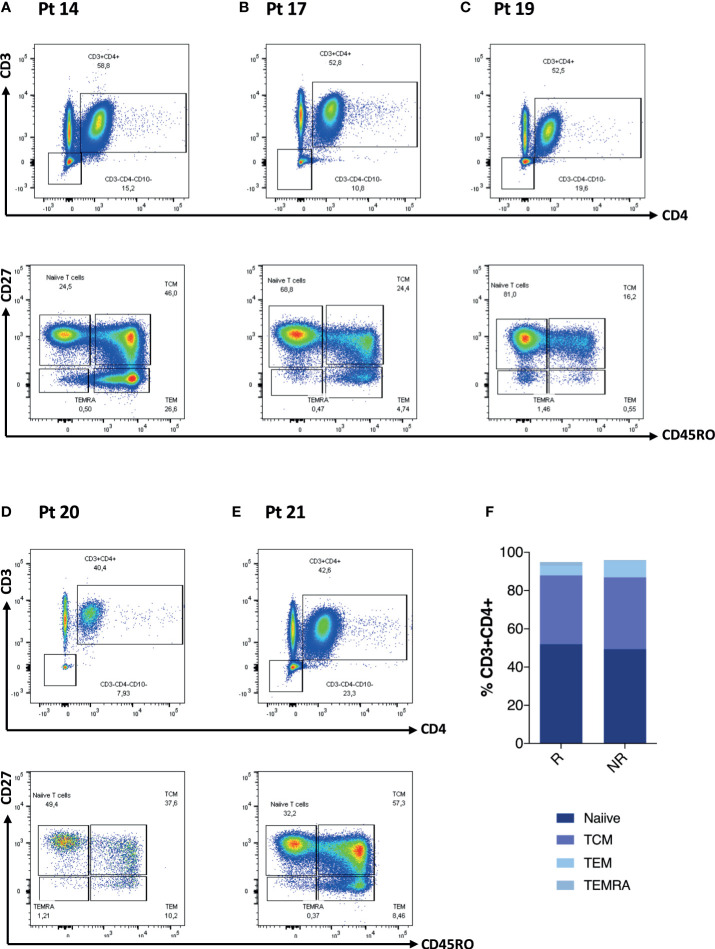
CD4 T-cell phenotype characteristics of five patients lacking any cellular response is reported in **(A–E)**. Non-paired non-parametric Mann–Whitney test was used to assess differences in the levels of each sub-population in **(F)**.

**Figure 3:**
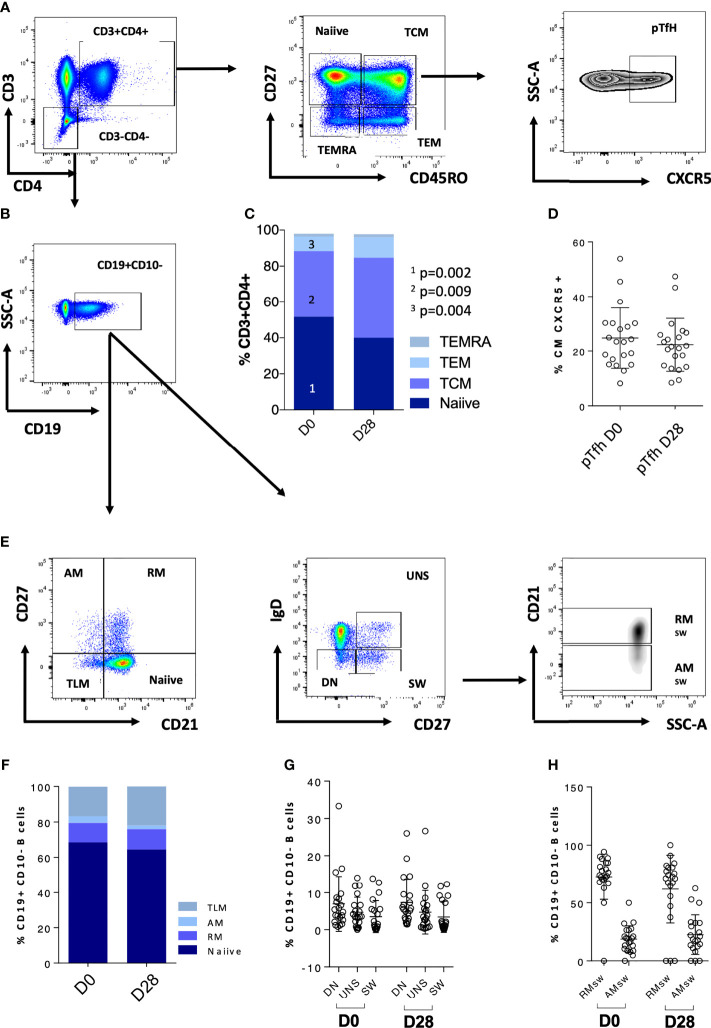
CD4 T-cell and B-cell phenotype characteristics of the whole cohort are reported. **(A)** Gating strategy for CD4 T-cell populations; **(B)** gating strategy for the CD19+ population used to analyze the B-cell subsets. **(C)** CD4 T-cell populations at D0 and D28; **(D)** pTfh at D0 and D28. **(E)** Gating strategy for the B-cell populations. **(F–H)** B-cell populations at D0 and D28. Non-paired non-parametric Mann–Whitney test was used to assess differences in the levels of each sub-population in **(C, D, F–H)**. TCM, central memory T cells; TEMRA, terminally differentiated T cells; TEM, effector memory; pTfh, peripheral follicular T cells; TLM, tissue-like memory; AM, activated memory; RM, resting memory; UNS, unswitched; DN, double negative; SW, switched; RM_sw_, resting memory switched B cells; AM_sw_, activated memory switched B cells.

We further explored CD4 T cells and CD4 memory subsets for these patients at D0 (**Figures 2A–E**). When analyzing these patients in comparison to the rest of the cohort, we did not observe any statistically significant difference in terms of frequency of T cell maturation subset (**Figure 2F**). We then explored the T- and B-cell phenotype for the entire IEI cohort (**Figures 3A–E**) and we observed changes in the frequency of T-cell memory subsets at D28 compared to baseline values. In particular, we observed a reduction of Naïve T cells (*p* = 0.002) in favor of an expansion of central memory (Tcm) (*p* = 0.009) and effector memory (Tem) (*p* = 0.002) (**Figure 3C**), following vaccination. On the other hand, frequency of peripheral T follicular helper cells (pTFH) (CD3+CD4+CD27+CD45RO+CXCR5+) did not vary at D28 (**Figure 3D**).

#### B-cell Response

We further explored the phenotypic maturation profile of B-cell subsets in IEI patients, and no variation of this compartment upon vaccination was found (**Figures 3E–H**). Overall, accordingly due to their immune impairment, these patients appeared to have very few switched B cells (mean = 3.4%, SD = 4.176%, **Figure 3G** and **Table 3**).

## Discussion

This work represents the first longitudinal immunological study on the efficacy of the BNT162b2 mRNA COVID-19 vaccine in a European cohort of 21 patients affected by IEI compared to HC. Our data show that patients with IEI are able to develop specific anti-spike antibody response in terms of both anti-trimeric S IgG and anti-S1-RBD IgG following vaccination, although at a significant lower magnitude (*p* < 0.0001) compared to HC (**Figures 1A, B**). The age was not associated with humoral response measured with anti-trimeric S antibodies or with the cellular response in both HC and IEI. On the other hand, we observed a weak significance between age and anti-RBD titers, indicating a lower humoral response in the older population compared to the younger one in HC, as widely documented by other groups (29–31). This result warrants a dedicated discussion because it does reinforce our findings. Indeed, despite the presence of an older group in HC that probably dragged down the median levels of Ab titer in HC, we were able to detect a weaker Abs response in IEI at D28. Long-term studies are needed to evaluate a potential early waning of vaccine-induced antibodies in IEI patients. Furthermore, we demonstrated that IEI are capable of sustaining generation of Ag-specific T cells after 1 week from the completion of the vaccination schedule.

Given that the robust elicitation of Ag-specific T cells represents the major correlate of mRNA-based vaccine efficacy (20), we further evaluated the SARS-CoV-2-specific T-cell response showing the ability of patients with IEI to increase the frequency of Ag-specific T-cell response upon vaccination. Of note, the CD4+CD40L+ T-cell subset was higher at D0 and D28 in IEI compared to HC as seen by the compensatory T-cell function in patients with primary B-cell defect (32). Despite the fact that the majority of IEI showed increased levels of Ag-specific T cells following vaccination, we observe that five patients failed to mount any cellular response (Pt14, Pt17, Pt19, Pt20, and Pt21), as usually observed in healthy individuals (20, 30), with Pt20 also lacking a specific humoral response. This rate of “non-responders” is in line with the only available study by Hagin et al. conducted on a similar cohort of Israelian IEI patients (33). We then explored to what extent this lack of response could be due to patients’ clinical condition. Pt19 and Pt20 are two siblings affected by a novel NFKB1 mutation (functional tests are in progress). Nuclear factor kB subunit 1 mutation represents one of the most common cause of CVID (34) with a wide range of clinical phenotypes (35–37). NF-kB is a key regulatory transcription factor involved in several aspects of the immune response including the development of specific immune responses (38).

Pt21 is affected by a heterozygous mutation of CTLA4 (c.G224A) and PTEN. PTEN is one of the major regulators of phosphoinositide 3-kinase (PI3K) signaling pathway playing a critical role in modulating T-cell activity (39, 40). In addition, CTLA-4 may also play a role in PI3K signals as well as in regulatory T-cell function (41), autoimmunity, and cancer (42, 43).

Altogether, these mutations could explain the impairment of specific T-cell response upon vaccination. For Pt14 and Pt17, a genetic diagnosis is not available yet.

Most of our patients have an immunodeficiency that mainly impairs the B-cell compartment. Our data show their relative ability to mount a specific humoral response upon two doses, although at a lower magnitude in comparison to HC. In this contest, the evaluation of Ag-specific T-cell response seems to be critically important to analyze vaccine-induced protection in this cohort. A discordant immune response as defined by the presence of humoral response in the absence of specific T-cell response was observed in roughly one-quarter of the IEI patients. These data raise immunologic concerns on the sole use of Ab response as a metric of protective immunity following anti-SARS-CoV-2 vaccine. Indeed, after natural infection, T-cell responses have been reported as a finer marker than Ab response (21, 44–46).

Patients with IEI are prone to develop persisting viral shedding, probably due to their impaired B- and/or T-cell function with subsequent higher risk of persistent viral replication and mutation within the host (47). Indeed, most variants were first described in immunocompromised patients (48). Moreover, specific immunomodulatory treatment could affect the immune response following vaccination (49). Taken together, these findings suggest that the evaluation of vaccine-induced immunity should also include quantification of Ag-specific T cells.

The following study limitations need to be mentioned: (a) the paucity of the sample size due to the Italian national regulation, which did not allow vaccine administration to patients living outside the Latium region where the two hospitals are located; (b) the short time of observation; (c) the lack of real-life data of protection against the different SARS-CoV-2 strains despite vaccination; and (d) variability of immune defects among subjects with IEI and within the same IEI condition.

In conclusion, our findings confirm the good safety and immunogenicity profile of the BNT162b2 mRNA COVID-19 vaccine in IEI patients and reinforce current national and international vaccine recommendation against COVID-19. The observation of an appropriate vaccine response in most patients should support trust on vaccination and immunization programs for distinct immune disorders. Studies of specific correlates to monitor persistence of vaccine-induced immunity will further support the design of tailored vaccine schedules for the benefit of these patients and the community.

## Data Availability Statement

The raw data supporting the conclusions of this article will be made available by the authors, without undue reservation.

## Ethics Statement

The studies involving human participants were reviewed and approved by Local ethical committee from Bambino Gesù Children’s Hospital and Policlinico Tor Vergata. Written informed consent to participate in this study was provided by the participants’ legal guardian/next of kin.

## Author Contributions

DA, AR, VM, and PP designed the project. AR and CP performed the experimental analysis. LC and CR performed Anti-SARS Cov-2 IgG Ab assays. DA, AR, and MS wrote the original draft. All authors contributed to the article and approved the submitted version.

## Funding

This study received funding from the Ministry of Health: RRC-2020-23669481 to NC and PP.

## Conflict of Interest

The authors declare that the research was conducted in the absence of any commercial or financial relationships that could be construed as a potential conflict of interest.

## Publisher’s Note

All claims expressed in this article are solely those of the authors and do not necessarily represent those of their affiliated organizations, or those of the publisher, the editors and the reviewers. Any product that may be evaluated in this article, or claim that may be made by its manufacturer, is not guaranteed or endorsed by the publisher.

## Supplementary Material

Supplementary Figure 1 Experimental approach to define SARS-CoV-2 specific CD4+CD40L+ T-cells. **(A)** Gating strategy for CD3+CD4+ T cells; **(B)** design and gating to analyze the CD40L+ T cells.

Supplementary Figure 2 Correlation between Age and humoral response or cellular response in HC and IEI.
