# Supplementary figures and images for: Humoral and Cellular Response Following Vaccination With the BNT162b2 mRNA COVID-19 Vaccine in Patients Affected by Primary Immunodeficiencies
Source: Front Immunol. 2021 Oct 4;12:727850. doi: 10.3389/fimmu.2021.727850 (PMC8521226; doi:10.3389/fimmu.2021.727850)

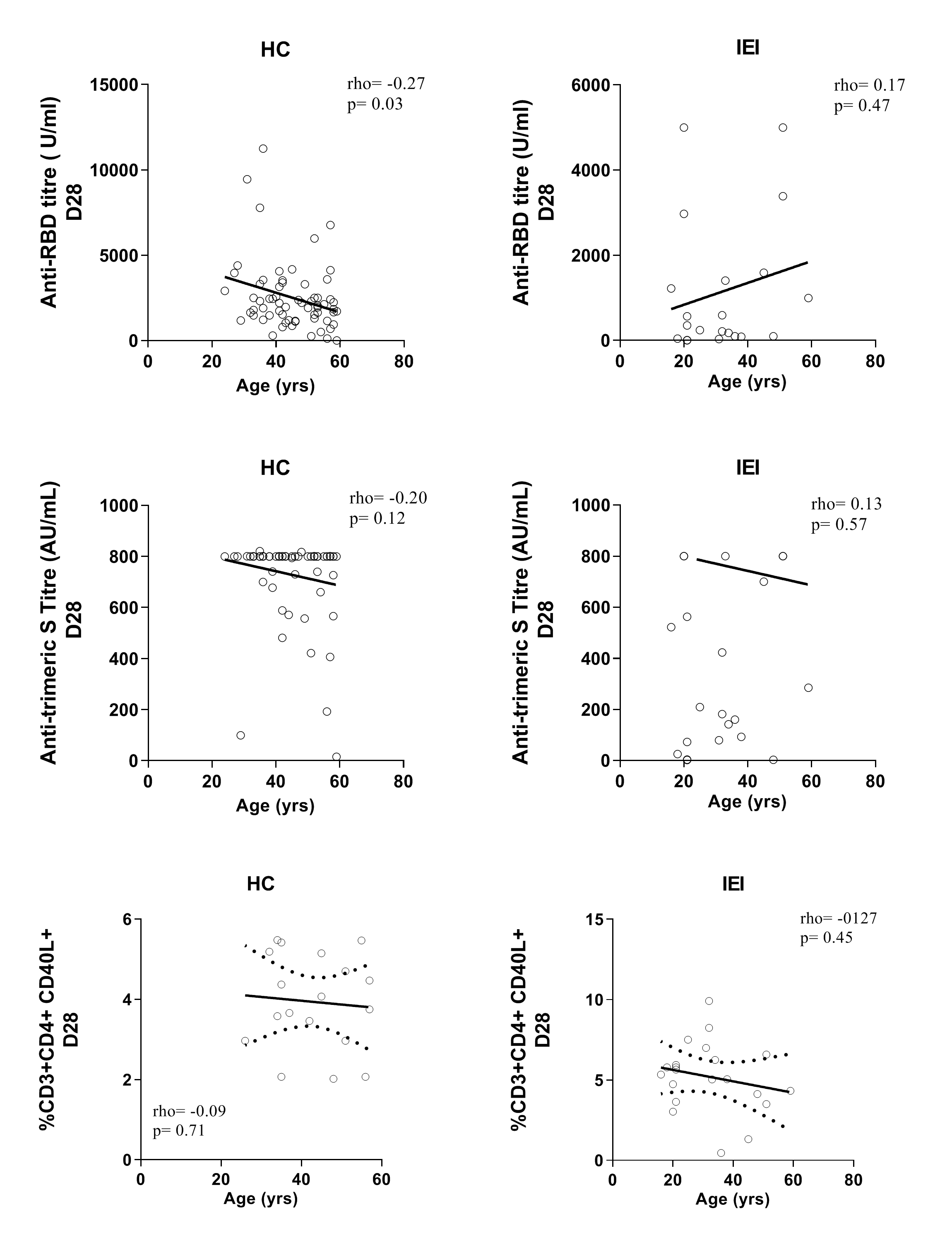

Supplement: Supplementary Figure 2 — Correlation between Age and humoral response or cellular response in HC and IEI. [file Image_2.tif]
